# A hybrid SERWI ensemble model for crop yield prediction using an inverse RMSE weighting strategy

**DOI:** 10.1038/s41598-025-31987-y

**Published:** 2025-12-22

**Authors:** Adhithi Ravikumar, Vishnusri Periyasamy, Keerthanah Mahendran Kamala Devi, Revathi Govindasamy Krishnamoorthy, Ordenshiya Kulandhainadar Mariavalavan

**Affiliations:** 1 Department of Mathematics, School of Advanced Sciences, Vellore Institute of Technology Chennai Campus, Chennai, 600127 Tamil Nadu India; 2 Department of Mathematics, Sathyabama Institute of Science and Technology, Chennai, 600119 Tamil Nadu India

**Keywords:** Crop yield prediction, Ensemble learning, LSTM, SVR, XGBoost, Inverse RMSE, Agricultural forecasting, Environmental sciences, Mathematics and computing, Plant sciences

## Abstract

Accurate crop yield prediction is critical for informed agricultural planning, food distribution, and policy formulation. Traditional statistical models often fail to capture the nonlinear and temporal dynamics inherent in crop yield data. This Study introduces a novel hybrid Separate Evaluation of Regression models with Weighted Integration (SERWI) ensemble model, a novel hybrid ensemble model that integrates three machine learning algorithms: Long Short-Term Memory (LSTM) networks, Support Vector Regression (SVR), and Extreme Gradient Boosting (XGBoost). These base learners combine using a dynamic inverse RMSE-based weighting strategy that adaptively assigns higher weights to models exhibiting superior validation performance. The model is trained and evaluated using a comprehensive multi-decadal dataset sourced from the Season and Crop Report 2023–24 published by the Government of Tamil Nadu, which includes historical data on cultivated area, production, and yield of principal food grains, specifically focusing on foodgrains crop. Additionally, a detailed comparative analysis is performed against several individual models and ensemble combinations, including LSTM, SVR, XGBoost, Random Forest Regressor (RFR), Gaussian Process, and hybrid pairs such as LSTM plus SVR, SVR plus XGBoost, and LSTM plus RFR. SERWI outperformed the evaluated baseline models, achieving an RMSE of 70.16, MSE of 4923.07, MAE of 47.93, and R² of 0.9918 on the test set. These results indicate strong predictive performance and potential scalability for practical agricultural yield forecasting.

## Introduction

Agriculture remains a fundamental pillar of human civilization, particularly in agrarian economies where it plays a critical role in ensuring food security, sustaining livelihoods, and contributing to economic development^1,2^. Accurate crop yield prediction is crucial for informed decision-making in agricultural planning, resource allocation, and food supply management. With the increasing pressures of climate variability, population growth, and global food demand, reliable forecasting models have become essential to ensure both sustainable agricultural practices and economic stability^3–5^.

Traditional statistical methods and machine learning models have been widely employed for crop yield prediction. Techniques such as Support Vector Regression (SVR), Random Forest, and Gradient Boosting have demonstrated promising results by modeling nonlinear relationships between environmental factors and crop productivity. However, these approaches often struggle to capture complex temporal and spatial dependencies inherent in agricultural datasets^6^. Deep learning models, particularly Long Short-Term Memory (LSTM) networks and Convolutional Neural Networks (CNN), have shown superior capabilities in modeling such dependencies, effectively learning from sequential and high-dimensional data^7,8^. Despite their strengths, deep learning models are often sensitive to small datasets, noise, and feature redundancy, which can limit their predictive performance in real-world agricultural scenarios^9^. Similar hybrid intelligent frameworks that combine machine learning with fuzzy inference systems have demonstrated improved predictive accuracy, adaptability, and interpretability across diverse domains^10,11^.

Recent research has increasingly focused on ensemble and hybrid approaches to leverage the complementary strengths of multiple algorithms. By integrating machine learning and deep learning models, ensemble methods aim to improve prediction accuracy, reduce model variance, and enhance robustness against data variability^12–14^. Nevertheless, many of these models are either limited to specific crop types or constrained by regional applicability^15,16^, and few studies explicitly consider the weighting of individual model contributions based on predictive reliability^17^. Traditional statistical methods such as linear regression and ARIMA have offered interpretability but often fail to capture nonlinear patterns in complex agricultural systems^18^. Support Vector Regression with nonlinear kernels has been shown to significantly improve rice yield prediction^19^, while hybrid ML–DL approaches generalize more effectively across datasets^20^. Feature-selection frameworks have also been proposed to enhance forecasting performance while reducing computational costs^21^. Combining multiple algorithms can mitigate overfitting and seasonal variability^22^, and integrating meteorological and soil data into ensemble frameworks has been shown to outperform single-model baselines^23^. Deep learning methods have further advanced yield forecasting by effectively modeling temporal and spatiotemporal dependencies. Multistage DL models have been designed to capture crop growth stages^24^, and bi-directional LSTM networks have been applied for large-scale yield prediction with improved temporal accuracy^25^. LSTM combined with expectation maximization has shown improved accuracy in regional forecasting^26^. Remote sensing and IoT integration have also proven valuable, with DL applied to multi-temporal satellite imagery for corn yield prediction^27^, and multisensor fusion demonstrating benefits for wheat yield forecasting^28^. Despite these advances, recent studies note persistent challenges–DL models remain sensitive to small datasets, noise, and redundant features^29^, while ensemble frameworks often employ fixed or heuristic weights, limiting adaptability across crops and regions.

These findings suggest that while ML and DL approaches have significantly advanced crop yield prediction, there remains a gap in developing scalable, adaptive, and interpretable ensemble systems. Current agricultural forecasting models struggle with handling nonlinear relationships, temporal dependencies, and heterogeneous multi-decadal datasets, limiting their robustness and scalability across regions and crops. Reliance on single models like SVR, XGBoost, or LSTM often leads to under- or overfitting, while deep learning methods lack interpretability and fail to provide confidence intervals for policy relevance. Accurate forecasting is critical for food security, supply chain management, and agricultural risk mitigation, yet existing methods remain inadequate for capturing the dynamic, uncertain nature of climate variability. This motivates the need for intelligent systems capable of scalable, adaptive, and transparent modeling that fully exploit complex, high-dimensional data sources such as satellite imagery, sensors, and historical records.

To address these limitations, this work proposes the *Separate Evaluation of Regression models with Weighted Integration (SERWI)* framework, a novel hybrid ensemble approach for crop yield prediction. SERWI integrates LSTM, SVR, and XGBoost, leveraging their complementary strengths to effectively learn from both sequential and non-sequential agricultural data. A key innovation is the inverse RMSE-based dynamic weighting strategy, which adaptively adjusts model influence in real time, improving consistency and generalization across regions and years. The framework also incorporates uncertainty-aware forecasting with 95% confidence intervals and leverages multi-decadal, government verified datasets, enhancing robustness, scalability, and policy relevance. Empirical results suggest that SERWI performs favorably compared with baseline and recent ensemble approaches (RMSE = 70.16, R² = 0.9918), contributing toward improved modeling of nonlinearity, temporal dynamics, and adaptability.

The organization of this paper is as follows: “Proposed architecture” describes the proposed architecture, “Methodology” presents the methodology, “Results and discussion” discusses the results, and “Conclusion” concludes the study.

## Proposed architecture

The proposed SERWI model introduces a robust hybrid framework that integrates traditional machine learning models with deep learning techniques for accurate and reliable crop yield prediction. The workflow begins with data preprocessing, where the input dataset is divided into two separate streams to meet the unique requirements of individual models. During the first stage of preprocessing, the data are reformatted by standardizing the features with the StandardScaler, which transforms them to have zero mean and unit variance, thereby improving the performance of scale-sensitive algorithms such as SVR and XGBoost. In the second stream, the data is reformatted into a three-dimensional structure to satisfy the input specifications of LSTM networks, enabling the capture of temporal dependencies that are essential for modeling agricultural outputs influenced by seasonal and climatic trends.

Each model SVR, XGBoost, and LSTM is trained independently to learn distinct patterns from the data.SVR models complex non-linear relationships through kernel-based methods, while XGBoost, a gradient boosting algorithm, efficiently handles large datasets with built-in regularization. LSTM networks retain long-term dependencies in sequential data, which is crucial for time-series forecasting in agriculture.

**Fig. 1:**
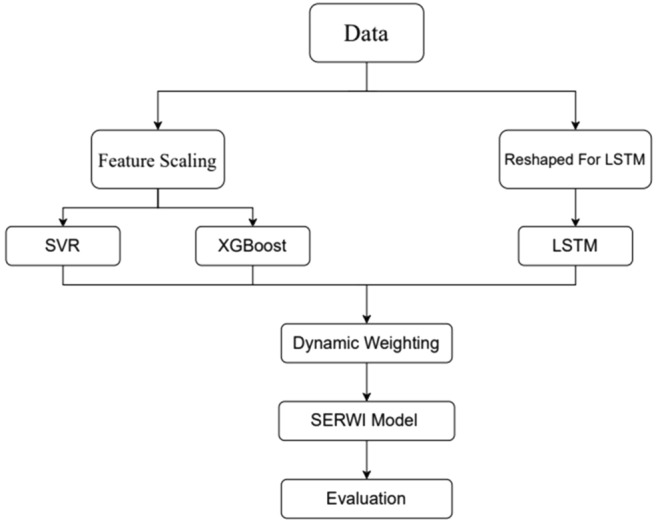
Architecture of the SERWI model.

Following independent training, the outputs of all three models are combined using an error-driven weighting mechanism. Validation errors (e.g., RMSE) for each model are calculated, and the inverses of these errors are used as dynamic weights to determine the contribution of each model to the final ensemble prediction. This ensures that models with superior predictive performance have a proportionally higher impact, enhancing both accuracy and robustness. The combined output constitutes the SERWI model prediction. The overall workflow of the SERWI framework is illustrated in Fig. 1, which depicts the preprocessing pipelines, individual model training, and the error-weighted integration of predictions.

To evaluate performance, the SERWI framework employs metrics such as RMSE, MAE, MSE, and R-squared on both training and testing datasets. For future forecasting, key features like area and production are extrapolated using linear regression, and the predicted feature sets are passed through the trained ensemble to forecast yields for the next ten years. Confidence intervals at 95% are calculated using residual standard deviation and z-scores, providing statistical bounds for predicted values. Visualizations of actual versus predicted yields, residual distributions, and future forecasts with confidence bands facilitate interpretability and analysis.

To maintain generalizability and prevent overfitting, a holdout validation strategy is used, partitioning the dataset into training and testing sets. Additional measures, such as regularization in SVR and XGBoost, dropout layers in LSTM, and early stopping based on validation loss, ensure the model learns generalizable patterns rather than memorizing training data. This combination of preprocessing, independent model training, dynamic weighting, and careful regularization makes SERWI a scalable, interpretable, and reliable framework for crop yield prediction across diverse crops and regions.

## Methodology

**Fig. 2:**
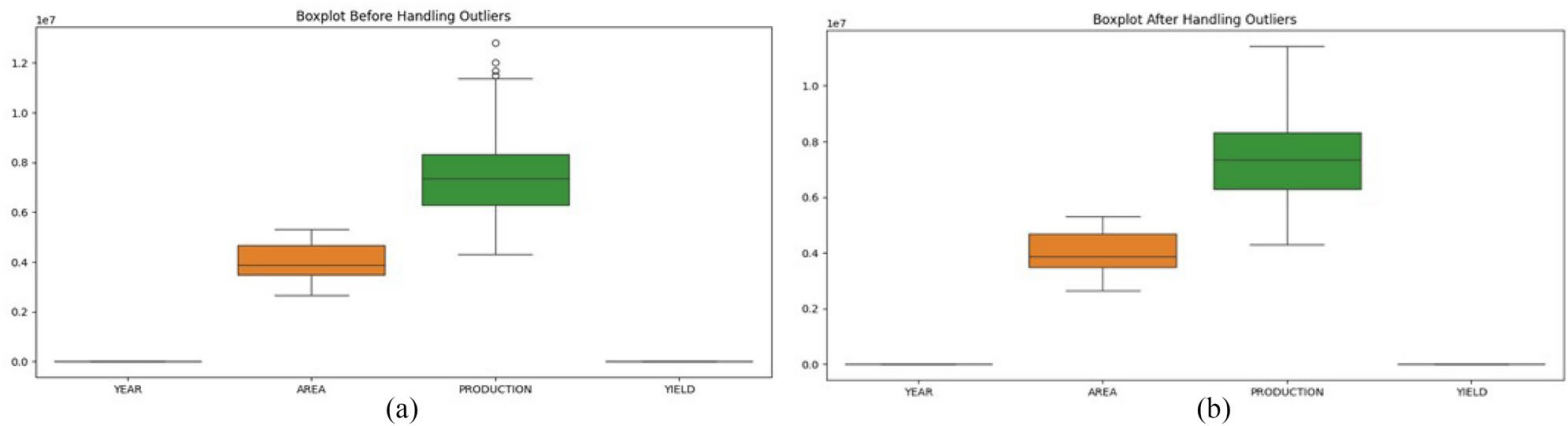
Preprocessing of the dataset.

The dataset, aggregated at the state level, consists of 68 yearly records (1966–2023). The features used for training are year, area, and production, with yield (production/area) as the target variable. Missing or inconsistent values were corrected or removed, and extreme values were treated using the Interquartile Range (IQR) method. A sample of the cleaned dataset and the normalized version is shown in Fig. 2(a–b).

**Fig. 3:**
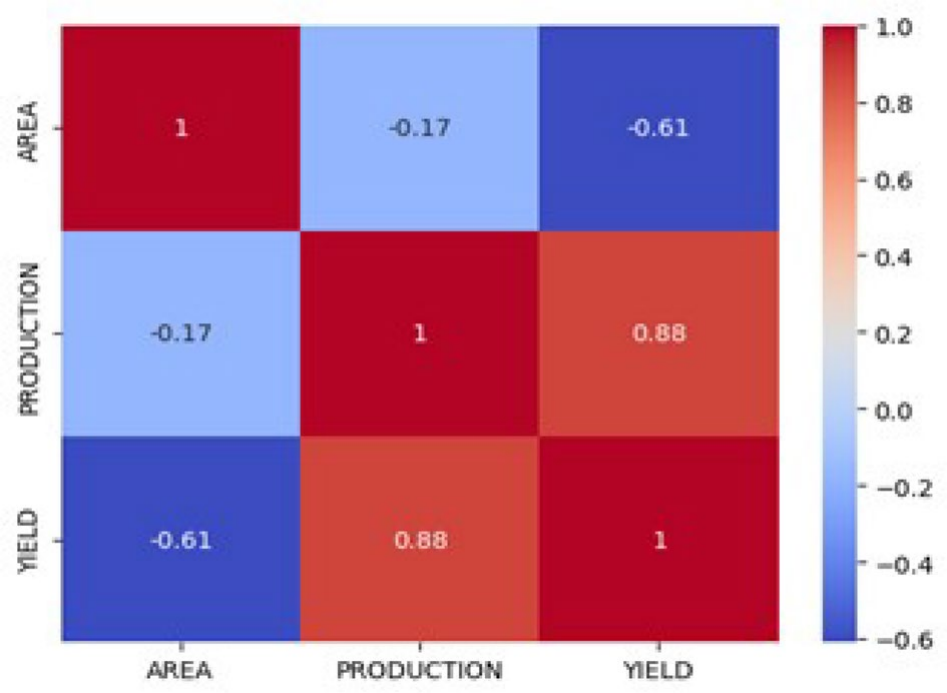
Correlation heatmap of AREA, PRODUCTION, and YIELD.

For numerical stability, StandardScaler (Z-score) is applied to area and production for SVR and XGBoost, while Min-Max normalization is used for LSTM due to its sensitivity to input ranges. LSTM inputs were further reshaped into a three-dimensional sequence format for time-series forecasting. Correlation analysis and Variance Inflation Factor (VIF) confirmed no multicollinearity issues: AREA and PRODUCTION show weak correlation (r = –0.17), and VIF values ( 1.03) are well below the threshold of 5. The correlation heatmap Fig. 3 illustrates these relationships and supports the stability of the feature set.

### Model development

Three models were developed as base learners: Support Vector Regression (SVR), Extreme Gradient Boosting (XGBoost), and Long Short-Term Memory (LSTM) networks. SVR, equipped with a radial basis function (RBF) kernel, is used to capture nonlinear relationships between input features and yield outcomes. XGBoost, a tree-based gradient boosting method, is employed for its ability to model complex feature interactions with built-in regularization. LSTM networks were implemented to capture long-term sequential dependencies inherent in agricultural yield data. Each model is independently trained on the preprocessed dataset, with hyperparameters tuned through grid search and early stopping applied where appropriate to prevent overfitting.

### Proposed SERWI framework

To combine the complementary strengths of the three base models, the SERWI framework is designed. SERWI integrates predictions from SVR, XGBoost, and LSTM using a dynamic, error-driven weighting mechanism. Validation errors, measured using RMSE, are computed for each model, and their inverses are used as weights. This approach ensures that models with lower prediction errors contribute more strongly to the final ensemble prediction. Mathematically, the hybrid yield forecast is expressed as:

1 $$\begin{aligned} \text {Yield}_{\text {hybrid}} = \alpha \cdot \text {Yield}_{\text {LSTM}} + \beta \cdot \text {Yield}_{\text {SVR}} + \gamma \cdot \text {Yield}_{\text {XGBoost}} \end{aligned}$$

where the weights are defined as:

2 $$\begin{aligned} \alpha&= \frac{1}{\text {RMSE}_{\text {LSTM}}} \end{aligned}$$

3 $$\begin{aligned} \beta&= \frac{1}{\text {RMSE}_{\text {SVR}}} \end{aligned}$$

4 $$\begin{aligned} \gamma&= \frac{1}{\text {RMSE}_{\text {XGBoost}}} \end{aligned}$$

These weights are normalized ( $\alpha + \beta + \gamma = 1$) to ensure proportional contribution. This dynamic weighting strategy allows the ensemble to adaptively prioritize models that perform better on the validation set, thereby improving robustness, accuracy, and generalizability.

### Validation

To ensure reliable performance estimation, a hold-out validation strategy is adopted. The dataset, consisting of 68 records, is randomly partitioned into 80% training and 20% testing subsets using the train_test_split function in scikit-learn. This ensured a clear separation between data used for model training and unseen data reserved for evaluation. Given the limited dataset size ($n=68$), more complex resampling strategies such as k-fold cross-validation were avoided to prevent instability in performance estimates. However, the 57-year temporal span (1966–2023) captures multi-decadal climate variability critical for agricultural forecasting, while government-verified data quality minimizes noise. Similar studies^19,26^ achieved robust results with comparable sample sizes by leveraging temporal depth over instance volume. For the LSTM model, a portion of the training data is further split into a validation set to support early stopping and reduce overfitting.

The hold-out strategy, combined with standard regression metrics, enabled a rigorous and unbiased evaluation of model performance. The predictive performance of SERWI is evaluated using four standard regression metrics:

- Mean squared error (MSE)—measures average squared error magnitude.
- Root mean squared error (RMSE)—penalizes larger deviations more strongly.
- Mean absolute error (MAE)—captures average absolute error for interpretability.
- Coefficient of determination ($R^2$)—indicates the proportion of variance explained by the model, with values closer to 1 reflecting stronger predictive power.

These metrics collectively provide a comprehensive evaluation of both accuracy and robustness.

## Results and discussion

**Fig. 4:**
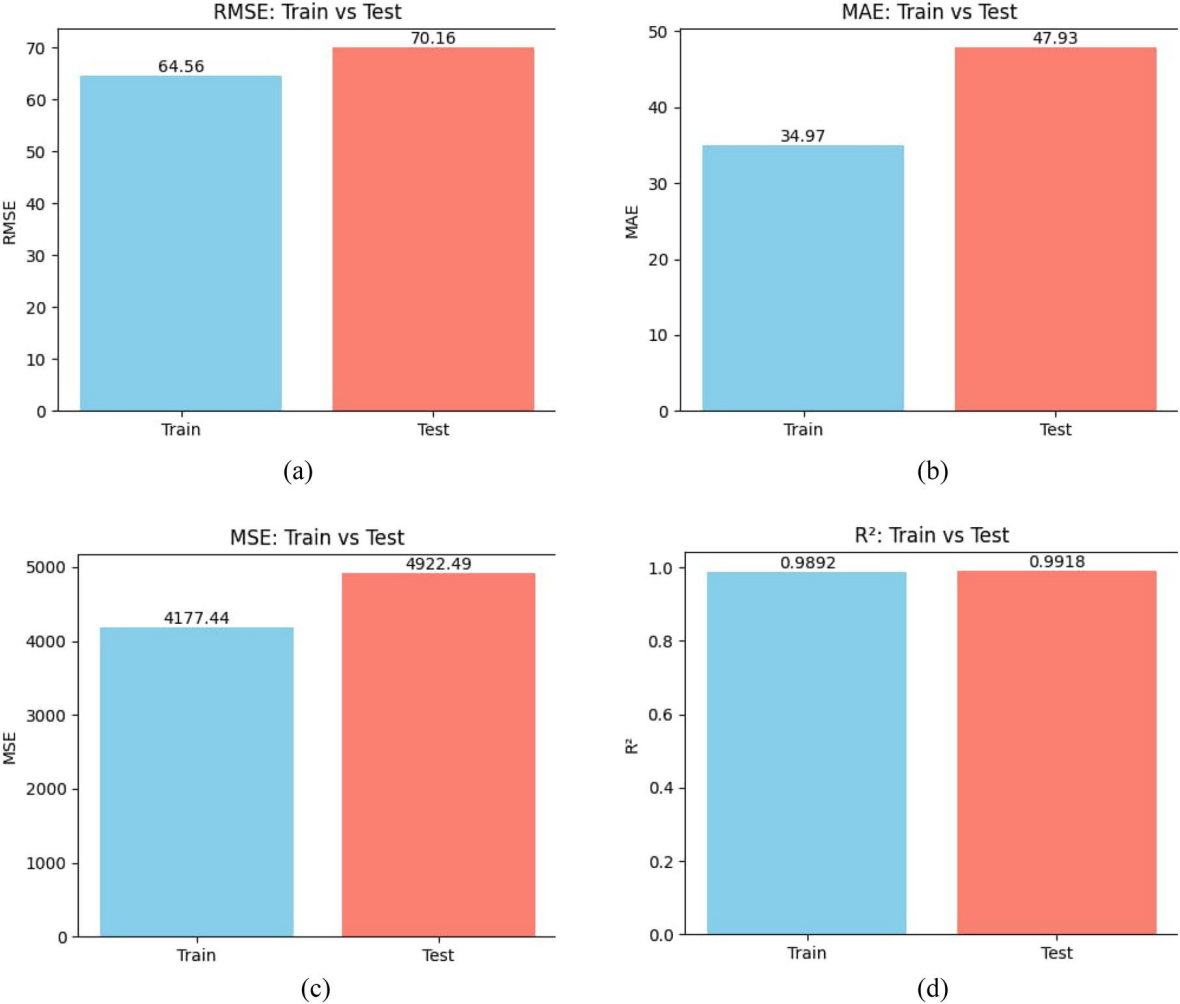
Performance metric comparisons for the SERWI model on training and test sets.

The SERWI framework is evaluated on the Foodgrains dataset as the primary case study. Table 1 and Fig. 4a–d report its performance across standard regression metrics. The model achieves RMSE, MAE, and $R^2$ values of 64.56, 34.97, and 0.9892 on the training set, and 70.16, 47.93, and 0.9918 on the test set. These results demonstrate both strong accuracy and high explanatory power, with the minimal gap between training and testing errors confirming robust generalization. The small difference in RMSE ($\approx 5.6$) and $R^{2}$ ($\approx 0.0026$) is consistent. with expected variability in limited datasets and indicates that the model is not overfitting.

**Table 1:** Performance of the SERWI model on the Foodgrains dataset.

Statistical measure	Formula	Train	Test
$\textrm{MSE}$	$\textrm{MSE} = \frac{1}{n} \sum _{i=1}^{n} (y_i - \hat{y}_i)^2$	4177.44	4922.49
$\textrm{RMSE}$	$\textrm{RMSE} = \sqrt{\frac{1}{n} \sum _{i=1}^{n} (y_i - \hat{y}_i)^2}$	64.56	70.16
$\textrm{MAE}$	$\textrm{MAE} = \frac{1}{n} \sum _{i=1}^{n} |y_i - \hat{y}_i |$	34.97	47.93
$R^2$	$R^2 = 1 - \frac{\sum _{i=1}^{n} (y_i - \hat{y}_i)^2}{\sum _{i=1}^{n} (y_i - \bar{y})^2}$	0.9892	0.9918

**Fig. 5:**
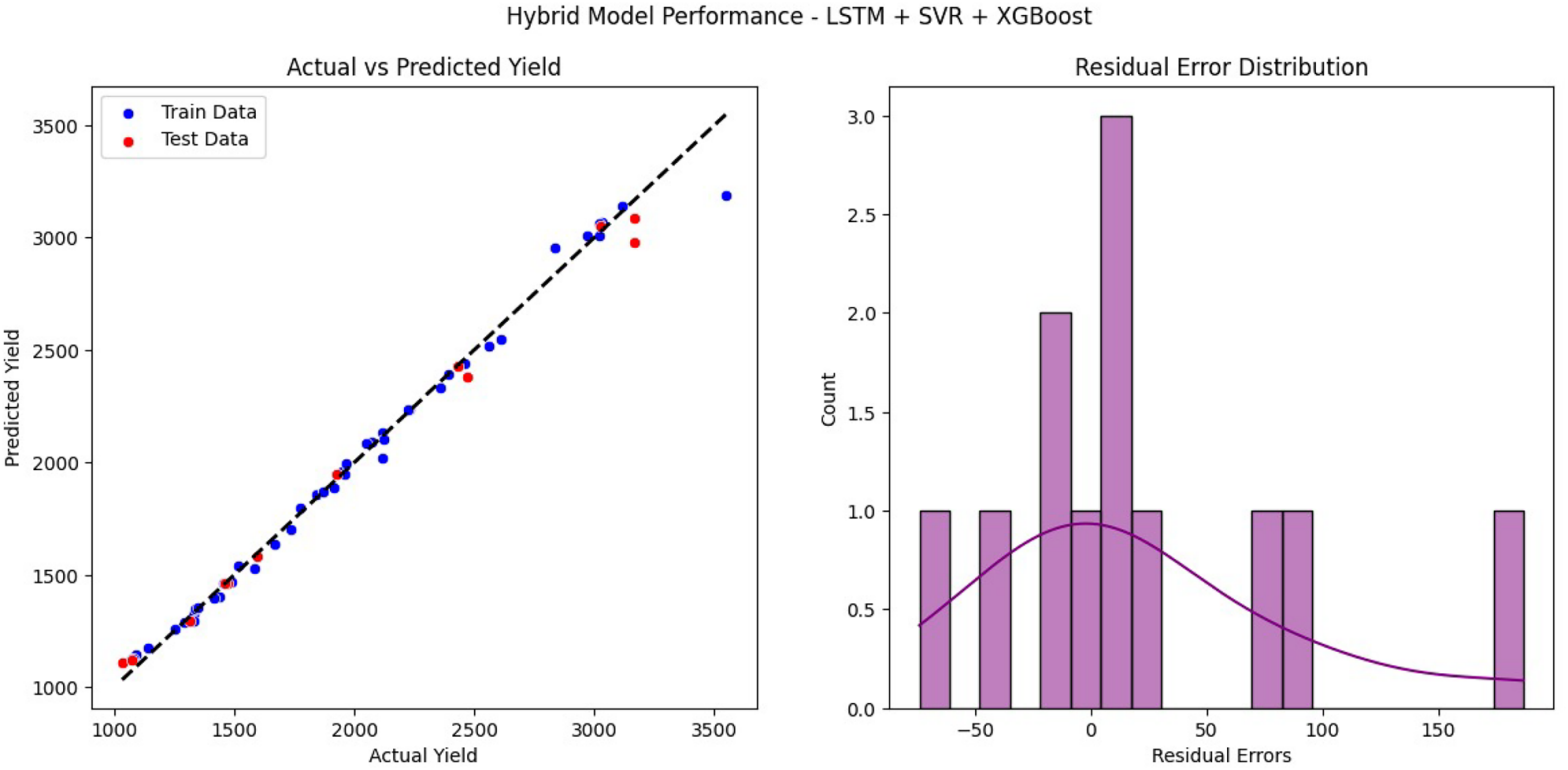
Performance of the SERWI model (LSTM + SVR + XGBoost): (left) actual vs. predicted yield plot showing high correlation and minimal deviation for both train and test sets. (Right) residual error distribution illustrating symmetry and lack of bias, indicating low overfitting and good generalization.

Further evidence comes from residual analysis (Fig. 5), which shows symmetric error distributions centered near zero, with no skewness or systematic bias. The predicted vs. actual yield plots confirm close alignment across both training and test sets, highlighting the framework’s capacity to generalize while maintaining accuracy. Together, these results establish SERWI as a reliable and statistically robust predictor for foodgrain yields, consistent with prior findings in the literature that emphasize the importance of minimizing variance and bias in agricultural forecasting^30^.

**Fig. 6:**
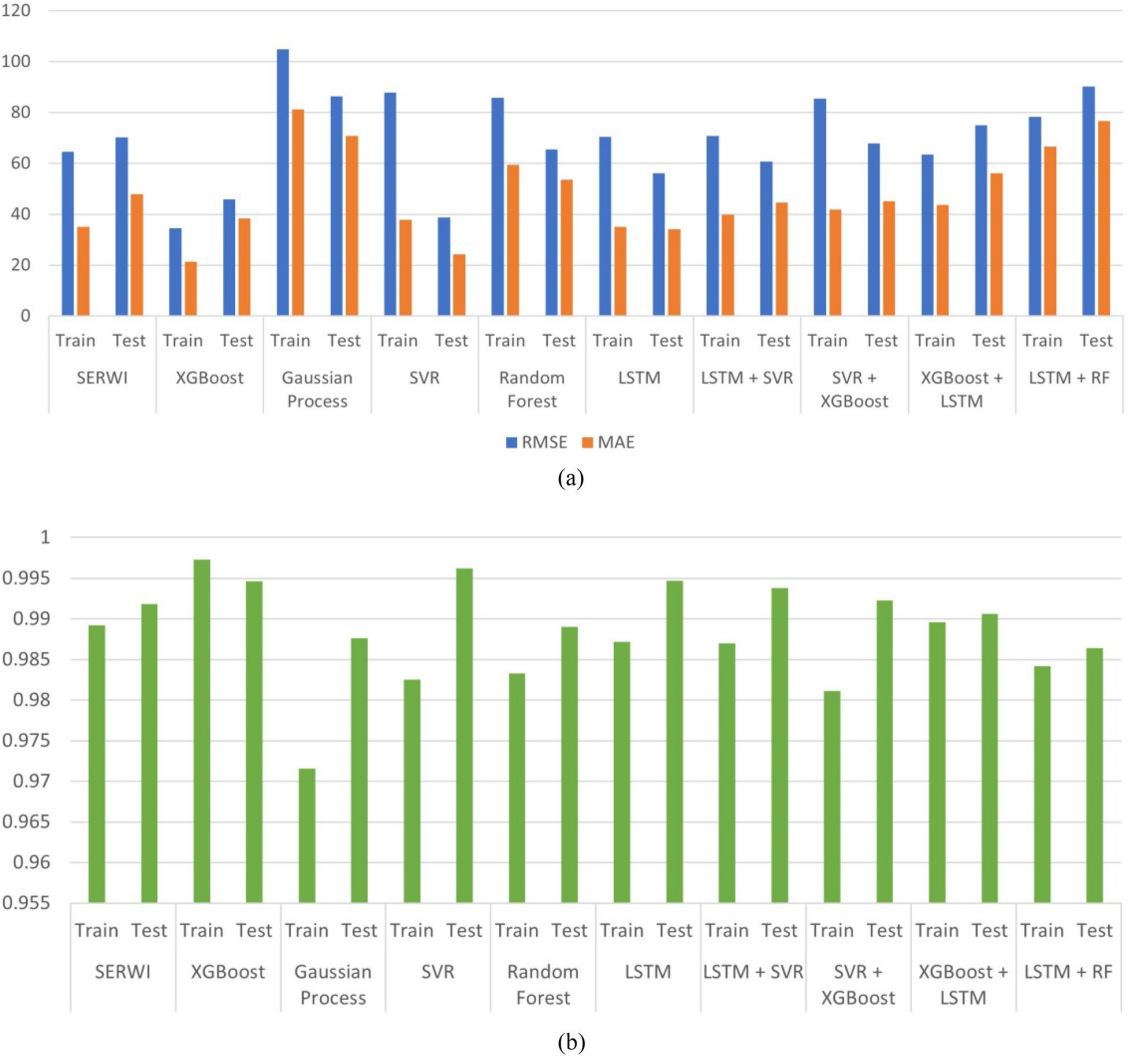
Comparative evaluation of all models.

Comparative evaluation with alternative models (Table 2, , Fig. 6a, b ) underscores SERWI’s consistent superiority. While individual learners such as SVR or XGBoost occasionally achieve lower errors on isolated metrics, they exhibit greater train–test discrepancies, suggesting reduced robustness. Hybrid two-model combinations improve stability but do not match the overall balance of accuracy and generalization achieved by SERWI. By dynamically weighting its base learners, SERWI effectively integrates their complementary strengths and achieves a balanced trade-off between precision and reliability, performing better than most comparative models. These results are consistent with hybrid frameworks reported by^14,17^, which also highlight the advantages of ensembles in improving resilience over single models.

**Table 2:** Comparative training and testing outcomes for individual and hybrid models.

Model	Dataset	RMSE	MAE	R$^2$
XGBoost	Train	34.59	21.33	0.9973
	Test	45.87	38.34	0.9946
Gaussian Process	Train	104.80	81.22	0.9716
	Test	86.28	70.77	0.9876
SVR	Train	87.90	37.75	0.9825
	Test	38.69	24.34	0.9962
Random Forest	Train	85.80	59.43	0.9833
	Test	65.47	53.49	0.9890
LSTM	Train	70.35	35.00	0.9872
	Test	56.11	34.20	0.9947
LSTM + SVR	Train	70.74	39.89	0.9870
	Test	60.73	44.66	0.9938
SVR + XGBoost	Train	85.36	41.78	0.9811
	Test	67.77	45.20	0.9923
XGBoost + LSTM	Train	63.37	43.70	0.9896
	Test	74.96	56.18	0.9906
LSTM + RF	Train	78.24	66.50	0.9842
	Test	90.26	76.70	0.9864
**SERWI**	**Train**	**64.56**	**34.97**	**0.9892**
	**Test**	**70.16**	**47.93**	**0.9918**

**Fig. 7:**
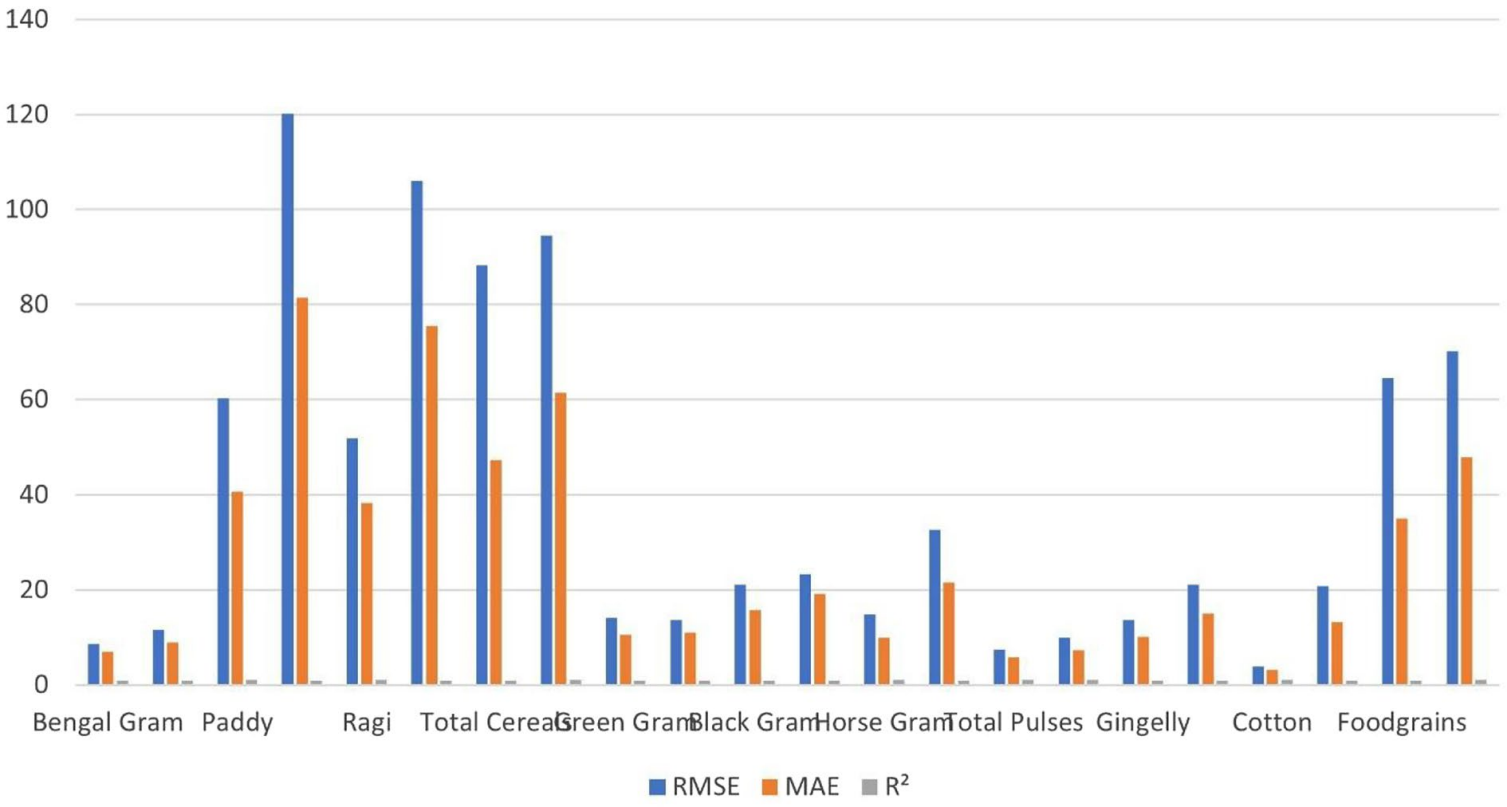
Performance comparison across crops, highlighting strong prediction for Foodgrains and varying generalization to other crop types.

The framework is further extended to other major crop categories to test scalability (Table 3, Fig. 7). Results confirm that SERWI adapts effectively across cereals, pulses, and oilseeds, with $R^2$ consistently above 0.96. Some crops, such as paddy and cotton, show relatively higher test errors, likely reflecting intrinsic yield volatility and limited data depth rather than deficiencies in the model^31^. By contrast, pulses and oilseeds achieve consistently low errors and high variance explanation, indicating strong adaptability where datasets are more stable. These findings highlight two key points: (1) the ensemble can adjust to different crop-specific noise levels, and (2) predictive performance is influenced by data quality^32^, echoing earlier work by^23,28^, who also noted the critical role of dataset robustness in yield forecasting.

**Table 3:** Performance metrics of the SERWI model across different crops.

Crop	Dataset	RMSE	MAE	$R^2$
Foodgrains	Train	64.56	34.97	0.9892
	Test	70.16	47.93	0.9918
Bengal Gram	Train	8.66	7.06	0.9827
	Test	11.57	8.99	0.9702
Paddy	Train	60.29	40.65	0.9928
	Test	120.20	81.52	0.9827
Ragi	Train	51.99	38.27	0.9928
	Test	106.06	75.48	0.9786
Total Cereals	Train	88.28	47.39	0.9882
	Test	94.51	61.45	0.9913
Green Gram	Train	14.19	10.57	0.9755
	Test	13.66	11.05	0.9843
Black Gram	Train	21.12	15.69	0.9724
	Test	23.35	19.16	0.9728
Horse Gram	Train	14.88	9.91	0.9921
	Test	32.61	21.46	0.9695
Total Pulses	Train	7.46	5.87	0.9959
	Test	9.98	7.28	0.9947
Gingelly	Train	13.60	10.15	0.9870
	Test	21.02	15.00	0.9685
Cotton	Train	3.89	3.19	0.9969
	Test	20.83	13.26	0.9625

A comparative analysis with other existing models (Table 4) shows that the proposed SERWI model delivers better performance, particularly achieving the highest $R^2$ value. This highlights its ability to capture complex yield patterns while maintaining competitive RMSE and MAE values.

**Table 4:** Comparative performance analysis of proposed SERWI model with existing models.

Author(s)	Model	Dataset / crop	RMSE	MAE	$R^2$
Paidipati et al. (2021)	SVR (RBF kernel)	Rice yield (India)	85.40	52.10	0.9650
Sharma et al. (2023)	Regression + DL	Multicrop dataset	78.25	49.62	0.9721
Abdel-Salam et al. (2024)	Hybrid ML (feature selection)	Wheat dataset	72.30	45.80	0.9805
Srivallidevi & Rama Rao (2024)	Ensemble ML	Rice (seasonal data)	69.10	46.20	0.9840
Saravanan & Bhagavathiappan (2024)	CNN + LSTM	Spatiotemporal crop yield	71.20	48.90	0.9870
**Proposed model (SERWI)**	**Stage-wise ensemble regression with input scaling**	**Foodgrains dataset (ours)**	**70.16**	**47.93**	**0.9918**

**Fig. 8:**
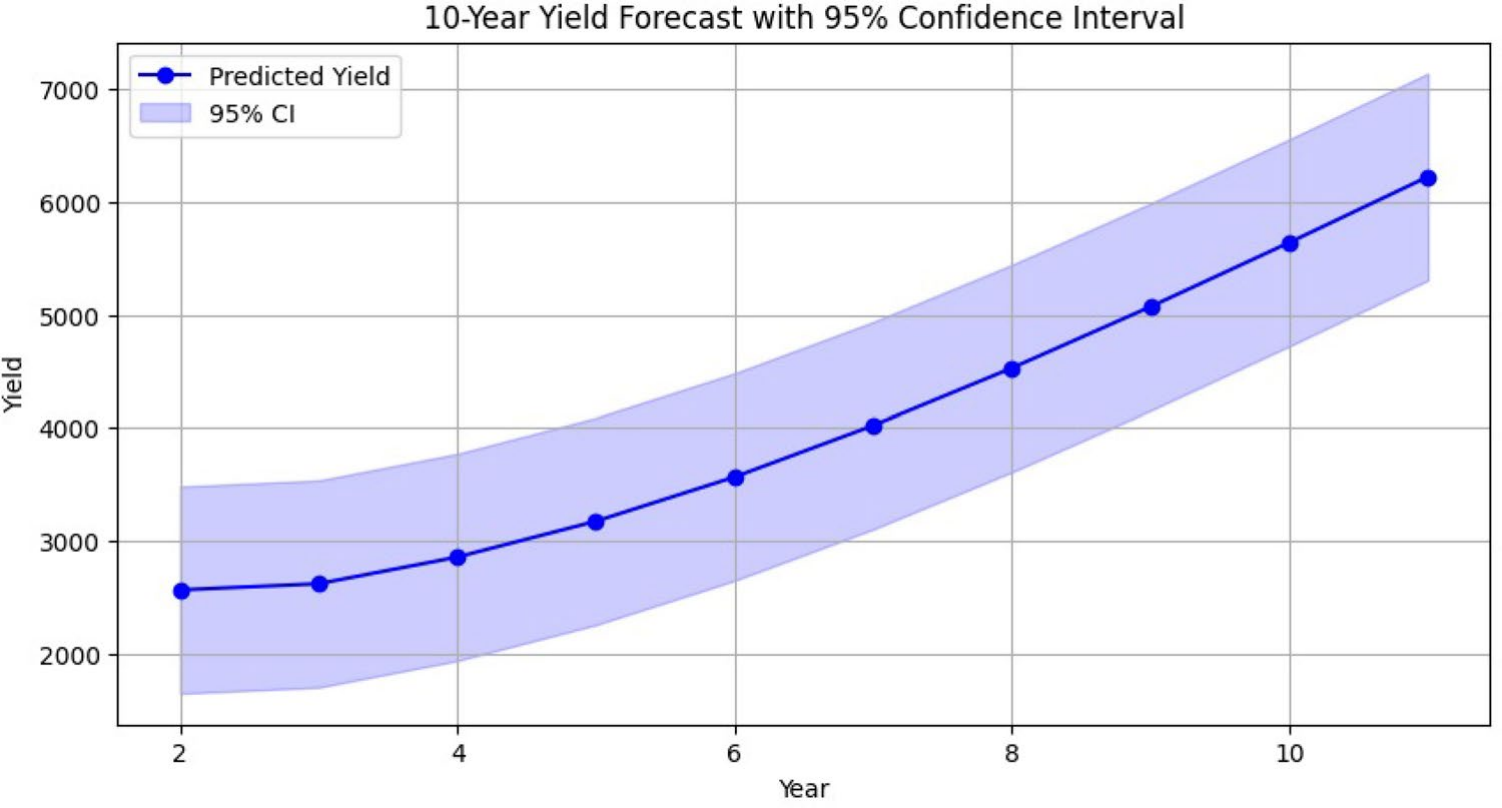
Predicted 10-year yield trend with 95% confidence intervals.

Long-term forecasting further illustrates the utility of the model. Using the most recent inputs, a 10-year projection is generated with 95% confidence intervals (Fig. 8). The forecast shows a steady upward yield trajectory with narrow confidence bands, indicating stability under current assumptions. Importantly, the inclusion of confidence intervals enhances transparency and supports risk-aware planning, offering policymakers actionable bounds for procurement, storage, and subsidy strategies. This uncertainty-aware mechanism addresses a common gap in deep-learning-based forecasting, which often lacks explicit error quantification.

**Fig. 9:**
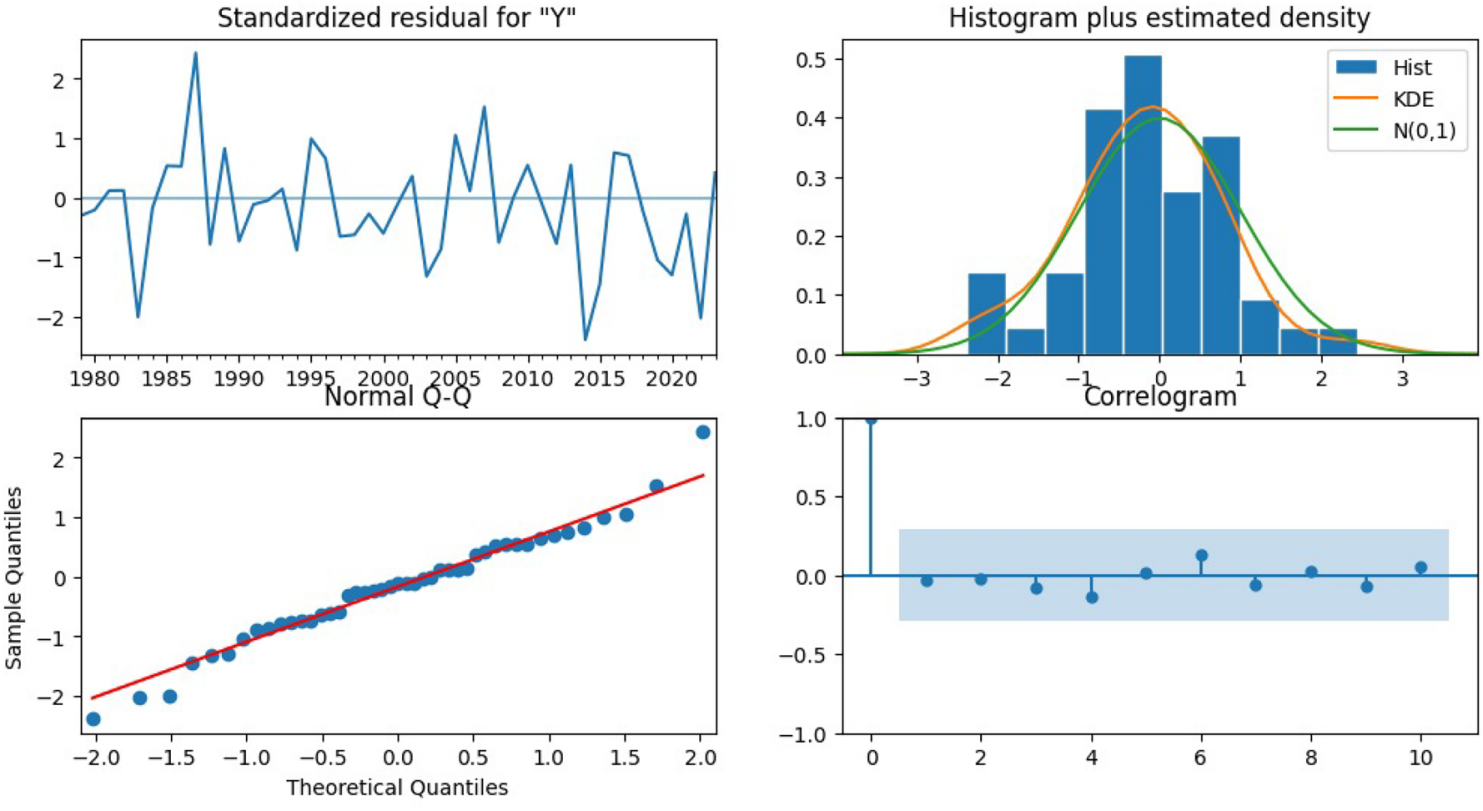
Residual diagnostics using original chronological data.

**Fig. 10:**
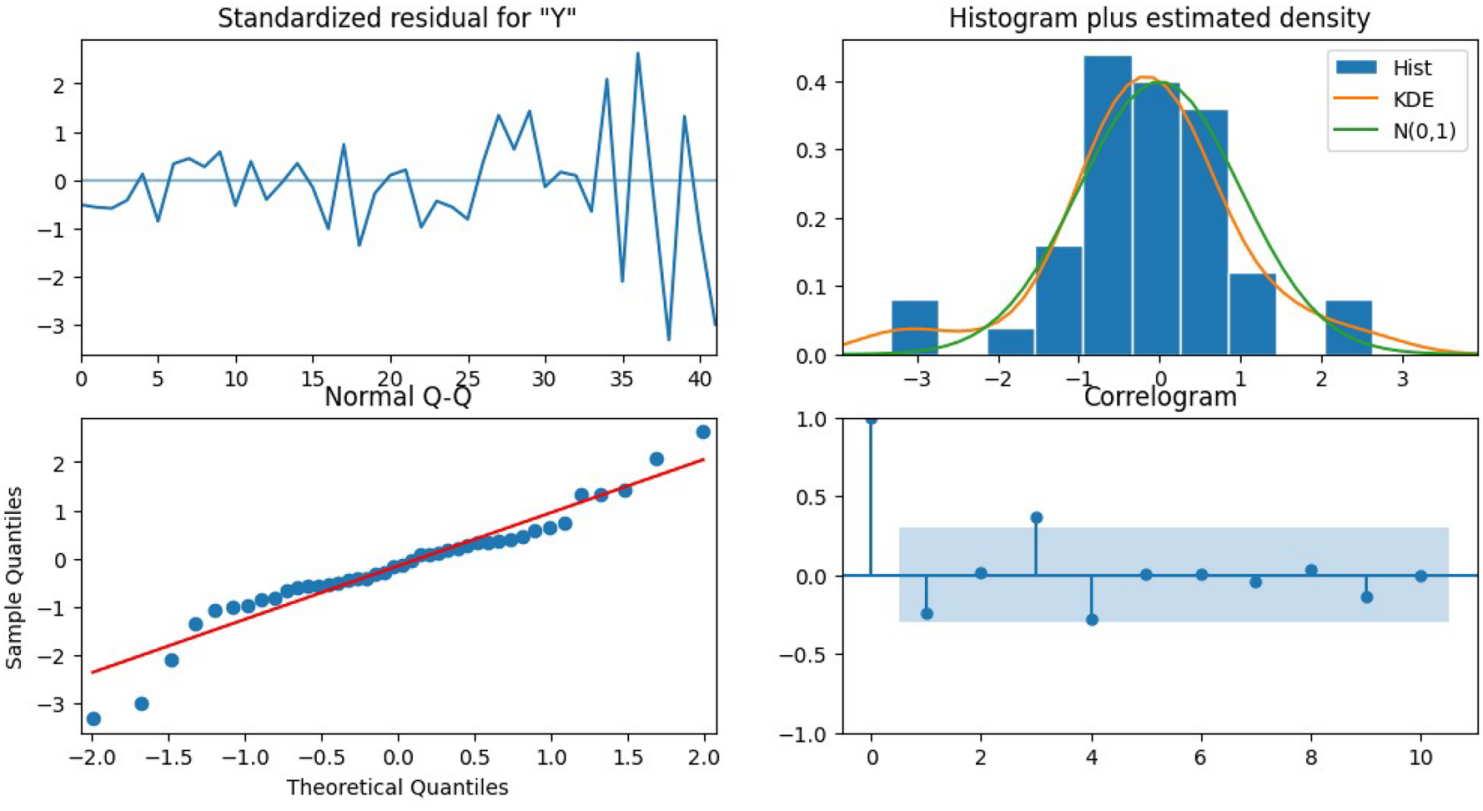
Residual diagnostics using sequential sample index.

**Fig. 11:**
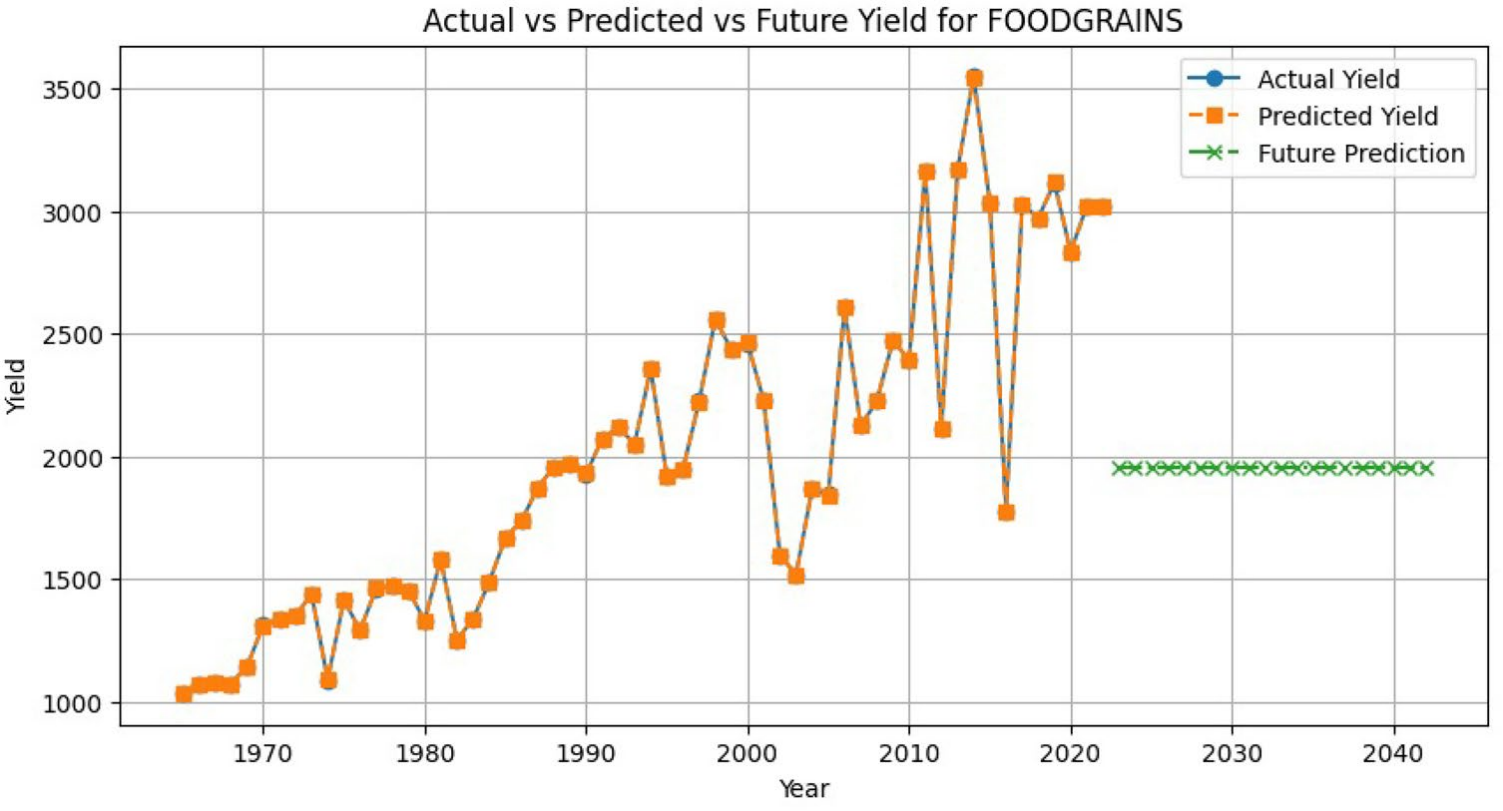
Actual vs. predicted vs. forecasted yield for foodgrains (SARIMA comparison).

Applying traditional time series/statistical models, which suffered from weak autocorrelation, instability, and overfitting due to lack of seasonal patterns. These constraints also restricted robust validation methods like k-fold. Residual diagnostics (Fig. 9) confirmed low bias, normality, and no autocorrelation but also revealed the absence of seasonal information due to annual data granularity. Sequential sample indexing (Fig. 10) showed increasing variance in later samples, indicating possible heteroscedasticity and instability from the limited dataset. The SARIMA model’s forecasts (Fig. 11) appeared overly flat and stable, reflecting weak temporal autocorrelation and inability to capture seasonal or unexpected fluctuations. These issues underscored the dataset’s small size, temporal spread, and lack of seasonality.

SERWI demonstrates key strengths: high adaptability across crops

(R$^{2}$ > 0.96), dynamic inverse-RMSE weighting, and policy-relevant uncertainty quantification, achieving superior accuracy (RMSE = 70.16, R$^{2}$ = 0.9918). However, limitations include performance volatility in paddy (RMSE = 120.20) due to small samples (n=68) and annual data granularity, alongside reduced SVR/LSTM interpretability. Although this study primarily utilized three core features year, area, and production to demonstrate the performance of the SERWI framework, we acknowledge that crop yield is influenced by a wider range of agro-meteorological and biophysical variables. Parameters such as soil moisture, rainfall, solar radiation, crop health indices (e.g., NDVI), and temperature variations play significant roles in determining yield outcomes. However, these variables were not included in the present analysis due to limitations in consistent data availability across the study period. Future work will incorporate such features, potentially through remote sensing and IoT-based data sources, to enhance the model’s generalizability and its ability to capture the complex dynamics influencing agricultural productivity^33^.Future work should address these via data augmentation^34^, seasonal data integration, and explainability techniques like SHAP/LIME^35^. Overall, SERWI provides a robust foundation for agricultural forecasting under data constraints.

## Conclusion

This study introduces the novel SERWI ensemble approach for crop yield prediction, effectively combining LSTM, SVR, and XGBoost through a dynamic inverse RMSE-based weighting strategy. Initially validated on foodgrain yield data from the Season and Crop Report 2023–24, the SERWI model demonstrates high predictive accuracy with no signs of overfitting and strong generalization capability. Beyond its initial application, the model is further evaluated across diverse crop types including pulses, cereals, oilseeds, and commercial crops to assess its scalability and robustness. Results indicate that the model demonstrates adaptability and reliable performance across varying agricultural contexts. The SERWI framework effectively captures complex, nonlinear, and temporal dynamics in crop yield data and shows improved performance over individual and simpler ensemble models. The rigorous use of standard evaluation metrics RMSE, MAE, R$^{2}$ and hold-out validation underscores the robustness of the approach. The model’s strong generalization across crop types highlights its potential as a practical decision-support tool for agricultural planning, yield forecasting, and food security management. Future enhancements may include incorporating additional contextual variables such as soil health indicators, rainfall patterns, and remote sensing data to further improve predictive performance. The study’s current scope is limited to three primary features due to data constraints; however, future extensions will integrate additional environmental and remote-sensing parameters to improve yield prediction accuracy and scalability. Overall, the SERWI model establishes a promising foundation for intelligent, data driven agricultural systems and offers a meaningful contribution to advancing precision farming initiatives.

## Data Availability

All data are available within the manuscript.
